# Enabling disability inclusive practices within the University of Cape Town curriculum: A case study

**DOI:** 10.4102/ajod.v4i1.157

**Published:** 2015-07-17

**Authors:** Chioma Ohajunwa, Judith Mckenzie, Theresa Lorenzo

**Affiliations:** 1 Disability Studies Programme, Department of Health and Rehabilitation Sciences, Faculty of Health Sciences, University of Cape Town, Cape Town, South Africa

## Abstract

**Background:**

Disability inclusion in the curricula of higher education institutions contributes to socially responsive graduates with a capacity to address the cross-cutting issue of disability in development. This article discusses a study conducted at the University of Cape Town (UCT), South Africa, to explore disability inclusion.

**Methodology:**

An instrumental case study approach was adopted and a thematic analysis of data was done.

**Findings:**

Academic staff found a variety of ways to include disability, such as discussions in class, practice and service learning, but mainly as part of disciplinary requirements. Including disability as an issue of social justice stems mostly from the personal interest of staff, and is done in an ad hoc manner.

**Conclusion:**

Disability should be valued, and integrated into the curriculum in a structured manner as a perspective on diversity with which to interrogate our beliefs about ourselves and society. Theorising on disability is needed, as well as the unique perspectives that emerge across interdisciplinary boundaries, especially within the African context.

## Introduction

‘Given the status of disability in our society, it may be that there needs to be larger recognition, or more formal recognition of that in the pedagogy’ (Participant 2:1). This opening quote from one of the study participants shows that the relevance of including disability in the curriculum has not yet been given the recognition it deserves. There are a variety of ways of understanding disability, but the two predominant models are the individual model of disability, and the social model. The individual model focuses on individual deficit or impairment, and attributes any restriction of activity or social disadvantage that the individual confronts in his or her everyday life as the inevitable and tragic consequence of that impairment (Hammell 2006). On the other hand, the social model (as proposed by the disability rights movement) posits that society creates barriers for any person with an impairment. These barriers include – but are not limited to – negative attitudes, and inaccessible environments, systems and structures. Disability arises when a person with an impairment is excluded because of societal barriers (Oliver 1996).

The social model has been widely adopted, albeit in varying forms, and underpins the United Nations Convention on the Rights of People with Disabilities (UNCRPD) (UN 2006), a human rights instrument intended to ensure equal participation and representation of people with disabilities in their communities. The UNCRPD has been widely ratified by member states. For the purposes of this study, we have adopted the definition of disability given by the UNCRPD (UN 2006), which views disability as an:

evolving concept, that arises from the interaction between persons with long-term physical, intellectual and sensory impairments and attitudinal and environmental barriers that inhibit their full and effective participation in society on an equal basis with others. (UN 2006:1)

The extent to which the environment in which the individual with an impairment operates is enabling or disabling is of primary importance and is thus foregrounded within this definition, as opposed to an emphasis on the impairment itself.

The UN has made specific recommendations regarding changes toward disability inclusion that should be led by universities and higher education institutions (HEIs) (Blumenthal & Boelen 2001). Article 8 of the UNCRPD, on awareness-raising, requires state parties to:

nurture receptiveness to the rights of persons with disabilities, and to promote positive perceptions and greater social awareness, fostering in all children and at all levels of education, the respect of people with disabilities. (UNCRPD 2006:11)

Article 24 of the UNCRPD, on education, strongly advocates the need to create awareness on disability issues in higher education, towards the support of lifelong learning for people with disabilities. There are a number of studies (Getzel 2008; Konur 2006; Lynch & Gussel 2001; Murray *et al.* 2009; Tagayuna *et al.* 2005; Vogel *et al.* 1999) that focus on strategies within HEIs for the inclusion of people with disabilities and the various ways they may be included, but the focus of this study is the wider impact arising from the inclusion of disability as a concept in the curriculum. HEIs have a unique position and influence which can be used to create a more inclusive culture, and the curriculum is one of the vehicles by which this change can occur.

For the purpose of this study, we adopted a broad understanding of ‘curriculum’, in three different ways. Firstly, the *intended* curriculum is concerned with the intended or overarching curriculum frameworks supplied by the discipline or institution for guiding what is taught to learners. Secondly, the *enacted* curriculum focuses on what is actually being taught in the educational institution; and thirdly, the *life* curriculum concerns meaningful classroom interaction (Marsh 2009). All three aspects of the curriculum are often influenced by the personal beliefs and understanding of the teacher. Chaney (2011) posits that the understanding one has of a concept impacts on ways of interacting with that concept; hence the relevance of exploring the understanding that lecturers have of disability inclusion in HEI curricula.

In this paper we begin by reviewing current international disability inclusion practices in HEIs; then, we discuss the methodology and study context, followed by the findings. Based on the findings, we argue that disability needs to be more firmly entrenched in the intended curricula of HEIs. We conclude by discussing the study implications and possible ways forward with regard to disability inclusion in HEIs.

### Current inclusion practices in higher education institutions

There are various arguments for the inclusion of disability in the curricula of HEIs, which include knowledge production, training of professionals, and the interdisciplinary nature of disability studies. In terms of knowledge production, there is increasing recognition that the role of HEIs is not only to provide access for students with disabilities, but also to build knowledge of disability into all academic spheres, and to produce graduates who are able to understand and deal with disability issues in their professional lives. Barnes (2007) advocates a change in knowledge production in higher education, so that disability becomes a cross-cutting issue that can influence the generation of new knowledge. This new knowledge occurs by disability opening up our thinking and helping us make sense of our existence and identities, identifying preferences and unconscious prejudices (Paetzold 2010). White (2004) states that disability inclusion in the curriculum generates new insights in teachers and students alike. Engaging with disability issues gives us a better understanding of ourselves, and helps us to interpret the experiences we have as human beings; Disability Studies helps society interrogate and understand who they are (Derby 2011).

The positioning of people with disabilities as ‘needing help’ has often been reinforced by societal perceptions, and impacts on how professionals are trained regarding disability. Transformation only occurs when default, long-standing beliefs are challenged (Chen 2014), and this needs to be considered in professional training. People with disabilities face major socio-political barriers, and some disability scholars advocate that one cannot engage with disability without considering history, gender and context, among other issues (Knoll 2009; Mawyer 2007). Learning about the context of policy implementation might address this need, and help transform service delivery for people with disabilities. Faculty staff and graduates who have undergone some disability training are more likely to provide reasonable accommodation for students with disabilities (Murray *et al.* 2009). An important element of this disability training would be to recognise that issues of disability cut across disciplines, and are not confined to the health and welfare professions.

Multidisciplinary inclusion draws on knowledge from different disciplines, but stays within their boundaries. The interdisciplinarity approach analyses, synthesises and harmonises links between disciplines into a coordinated and coherent whole. Transdisciplinarity integrates the natural, social and health sciences in a humanities context, and transcends their traditional boundaries. The objective of multiple disciplinary approaches is to resolve real-world or complex problems, to provide different perspectives on problems, to create comprehensive research questions, to develop consensus on clinical definitions and guidelines, and to provide comprehensive health services. Multiple-disciplinary teamwork has both benefits and drawbacks (Choi & Pak 2006).

The notion of an interdisciplinary disability studies would integrate the contributions of various disciplines to a problem, issue or theme related to disability. Various researchers would also work together to transfer knowledge related to disability between disciplines, while retaining their discipline-specific methods (Rebbeck, Paskett & Sellers 2010).

The transdisciplinary approach is the most complex level of integrated study, but often contributes to societal change (Derby 2011; Meeth 1978, cited in Rebbeck *et al.* 2010). However, although this study advocates a transdisciplinary approach as the ideal, it also aims at fostering space for an interdisciplinary approach to disability inclusion.

This interdisciplinary approach would include disability as an issue of social justice and diversity, and would invite various understandings of disability as linked to all disciplines of knowledge. This meaning-making across all disciplines would greatly enhance the inclusion of disability in the curriculum. Disability has been successfully included in the humanities and the built environment curricula in some HEIs (Danso, Owusu-Ansah & Alorwu, 2012; Derby 2011; Kanter 2011). The fact that disability is included in various disciplines indicates that full knowledge on disability does not reside within one discipline only (Campbell 2009; Gabel 2010).

HEIs are beginning to include disability issues in their teaching and research, employing various methods and strategies of inclusion (Strauss & Sales 2010). Previously, the response of HEIs to disability was directed largely towards increased access for disabled students. The effect of disability training on faculty attitudes has been identified by various studies as important (Getzel 2008; Konur 2006; Mayat & Amosun 2010; Murray *et al.* 2009; Vogel *et al.* 1999). For example, a study done in the Faculty of Engineering and the Built Environment at the University of Cape Town (UCT) showed that faculty staff are willing to accommodate disabled students, but struggle with their limited knowledge relating to the accommodation of disabled students, as the lecturers themselves had received no prior disability-related training within their own discipline (Mayat & Amosun 2010). This illustrates the need to begin to give time and space in current HEI curricula to include disability in a more structured manner, so that the knowledge base of lecturers can be expanded to facilitate inclusion.

There are some examples of HEIs that already include disability in their curricula. The University of New South Wales in Sydney has a disability-inclusive theoretical and philosophical framework as part of their social work undergraduate curriculum (Meekosha & Dowse 2007). In the field of education, it is advocated that teachers be critical of their reactions to disability, and be aware of any internal prejudices they may have about disability (Ware 2008). Disability is included in the Art curriculum of the University of Kansas, for the purpose of understanding and transforming issues of oppression (Derby 2011).

A range of strategies has been used for disability inclusion. Practice/service learning or experiential learning sessions were identified as the most prevalent method of disability inclusion (Campbell 2009). In this strategy, students are exposed to diverse social contexts, where they work and interact with people with disabilities in a bid to understand the lived experience of those people. This particular strategy has been criticised as inadequate; because the focus is on experiential learning, students often do not gain the knowledge of various theoretical concepts related to disability. The challenge of addressing theoretical aspects of disability inclusion could be attributed to a lack of time available in the curriculum for this purpose (Silver, Bourke, & Strehorn 1998).

It is hoped that the inclusion of disability in curricula would ensure that students are equipped to gain knowledge of the complexities of this global issue. Inadequate preparation of HEI graduates – future leaders, who will contribute to and work with people with disabilities – results in great injustice. This study therefore aimed to explore the understanding and practice of academics across a range of disciplines with regard to disability inclusion in curricula across all faculties at UCT.

## Methodology

A qualitative instrumental case study approach was adopted. Academic staff from all six faculties at the University were interviewed. In-depth, face-to-face interviews guided by prompts were conducted, providing a rich source of data (Silverman 2001:114). The interview guide was developed from the research team's knowledge and experience of the research area unstructured discussions with people who have personal experience of the research area, and a review of the literature (Cassell & Symon 1994).

### Study context

As an HEI, UCT has various policy frameworks that guide procedures, structures, and the implementation of programmes and services. A number of these policies are aimed at creating a more inclusive institutional culture that will enhance diversity and tolerance of difference in the University. The Vic Chancellor developed six strategic goals as part of the strategic plan to develop UCT (2009) in particular ways over 2010–2014. The two strategic goals of *expanding and enhancing UCT's contribution to South Africa's development challenges* and *enhancing the quality and profile of UCT's graduates* were selected as the focus of the study, due to their relevance to the issue of disability inclusion in teaching, learning and research across all disciplines. UCT's governance structures, which include the Transformation Office, drive these policies, and faculties are held accountable for ensuring that social responsiveness is included in their portfolios (UCT 2012).

UCT has approximately 26 000 students across the six faculties, the Centre for Higher Education Development (CHED) and the Graduate School of Business (GSB). The six faculties are Engineering and the Built Environment, Health Sciences, Humanities, Sciences, Law, and Commerce; they have approximately 60 departments between them, including associate departments and programmes. The Disability Services Unit provides support and reasonable accommodation to disabled staff and students, to assist their functional capacity at the University. The Disability Studies Programme (DSP) in the Department of Health and Rehabilitation Sciences (Faculty of Health Sciences) is the academic programme that housed this study.

### Sampling and inclusion criteria

#### Ethical clearance

Academic staff who include disability in teaching were interviewed, identified through a questionnaire circulated to all the faculties. After the interviews, some participants identified other staff who include disability in teaching. The researcher then contacted and interviewed these additional staff members. A total of 42 academic staff from all six faculties participated in the study.

Data was gathered using in-depth, face-to-face interviews, using an interview guide. Each interview lasted approximately an hour. Interview transcripts were transcribed verbatim; then, each transcript was read repeatedly to gain familiarity with the data. A thematic analysis was done to identify themes that emerged relating to disability inclusion.

Participants gave their informed consent for the interview, and agreed to being audiotaped. Ethical approval for the study was received from the Faculty of Health Sciences Human Research Ethics Committee, with approval number HREC REF: 653/2012.

## Findings

Four themes emerged, and will be expanded on:

- motivation for disability inclusion
- understanding of disability
- focus of inclusion
- teaching strategies.

## Motivation for disability inclusion

The theme of motivation for disability inclusion relates to the reasons that participants gave for disability inclusion. ‘Disciplinary requirement’ refers to the intended curriculum, as given by the discipline or department; ‘influence of a colleague’ refers to participants who were influenced by colleagues; while ‘personal interest’ refers to participants who included disability because of their own interest. It was revealed that participants include disability mainly because it is part of their disciplinary requirement (Table 1).

**TABLE 1:** Motivation for disability inclusion.

Variable	Health Sciences	EBE	Humanities	Law	Commerce	Science
Disciplinary requirement	11	4	3	3	1	1
Influence of a colleague	0	0	1	1	0	0
Personal interest	5	5	7	0	1	1

The dominant motivation for disability inclusion in the curricula of the Faculties of Health Sciences, Science, Commerce and Law relates mainly to disciplinary requirements, followed by the personal interest of the lecturer.

‘My personal approach is inclusion, enablement, the social model. However, the department takes a medical approach; so disability issues do not come in as a formally integrated aspect of the teaching, but individuals bring that aspect in.’ (Participant 1:8)

In the Faculty of Health Sciences, the priority is to fulfil the curriculum requirements for teaching of the Health Professional Council of South Africa Board. This results in a greater focus on impairment, if the social justice perspective is not originally part of the departmental curriculum. Some departments include disability only if related to a topic under ad hoc discussion. Responses from the Faculties of Law and Commerce showed that disability is included when any legislation or policy considered in class discussions is linked to disability.

‘In fact, disability is used as an example of what we call a Collective Right, rather than a Corporate Right; but it's not the main focus. It's merely an example.’ (Participant 4:2)

The Faculty of Sciences included disability in terms of using technology to enhance the quality of life of people with disabilities. In the Faculty of Humanities, ‘personal interest’ was the dominant motivation for including disability.

### Understanding of disability

The second theme describes the ‘lenses’ through which participants view disability. Although the influence of both the individual and the social models of disability could be seen across most faculties, the individual model was the most predominant in understanding disability (Table 2). ‘Disability in architectural terms is mainly around universal access. That's the first, primary concern, because access is being able to get to all parts of the building’ (Participant 2:1).

**TABLE 2:** Understanding of disability.

Variable	Health Sciences	EBE	Humanities	Law	Commerce	Science
Individual model	12	3	3	3	2	1
Social model	4	3	5	0	1	0
Biopsychosocial	3	0	0	0	0	0
Developmental	0	0	1	0	0	0

The Faculty of Law focused on mental impairment and how this impacts on an individual during a judicial process, while the Faculty of Humanities focused almost solely on a social model of disability, including the socio-cultural causes, resources and impacts of disability. This focus can be attributed to the predominant viewpoint that although one may have an impairment, socio-cultural and familial context has more of an impact on how the individual experiences the disability.

‘Disability is contextual and cultural and familial and personal. So it… so somebody with a perceived disability could have been raised in a family where it was not perceived as a disability; and experience, you know, great opportunities and conditions for possibility.’ (Participant 3:1)

Although the Faculty of Engineering and the Built Environment recorded the use of both the social and the individual model of disability, statements from that faculty indicate that the extent of inclusion needs to be reviewed. ‘Given the status of disability in our society, it may be that there needs to be larger recognition, or more formal recognition, of that in the pedagogy’ (Participant 2:1)

In the Faculty of Health Sciences, the Department of Health and Rehabilitation Sciences offers a curriculum rich in disability content. The divisions of Disability Studies, Occupational Therapy, Communication and Speech Disorders, Physiotherapy and Nursing provide a wider understanding of disability as a human rights issue, using both the individual and social models of disability, and the biopsychosocial and developmental approach.

### Focus of inclusion

The third theme addresses curriculum content taught to students, with the focus ranging from impairment, to disability as an issue of diversity (as with gender and race), to human rights, to involving students in discussions on the theoretical and policy contexts of disability. Impairment, human and socio-political rights, and issues of access were the main areas of teaching focus related to disability across all faculties (Table 3).

**TABLE 3:** Focus of inclusion.

Variable	Health Sciences	EBE	Humanities	Law	Commerce	Science
Impairment/health conditions	10	0	3	1	1	0
Diversity/enablement	1	0	3	0	0	0
Human/socio-political rights/access	11	4	7	1	1	1
Disability management, Community and family participation/ CBR	4	0	1	0	0	0
Gender, intersectionality, poverty, oppression	2	0	4	0	0	0
Disability related policy and legislature	2	1	0	1	1	0
Developmental issues related to disability	2	1	1	0	0	0
Theoretical discussions	1		1	0	0	0

The Faculty of Health Sciences focuses mainly on the preventive, curative and rehabilitative aspects of impairment. In the Department of Health and Rehabilitation Sciences, disability concepts are taught at undergraduate level as well as at postgraduate level, where students are also encouraged to produce full dissertations on disability-related issues. Opportunities are created to encourage experiential learning about disability.

‘Some of the programmes actually have community placements, where they can actually see – what rehabilitation is required, how the communities adapts, how the families adapt… students follow families through from the ICU to the wards, and then three family visits. They not only access how the patient is doing, but how the family is coping.’ (Participant 1:12)

In the Faculty of Humanities, students from the Social Development Department are encouraged to explore how the position of a breadwinner in the family can be changed by disability, and the resultant mental strain to that individual. At the postgraduate level there is a focus on disability as a development issue; various policies on social development relating to disability are explored, and presentations are made by students:

‘Whoever was interested in the topic of disability and development worked then in that one small group, with many other small groups on different other aspects, and then would read up about the latest legislation and (international, down to local) policy, and NGOs, and put across the challenges to integrating disability into development.’ (Participant 3:2)

In the Faculty of Engineering and the Built Environment, disability is included in terms of compliance with legislature and issues of physical access, so there is a lot of focus on physical disability and how structures can accommodate ‘difference’:

‘The main area is design, and – in a sense – configuring space for human activity. So it's integrated, in the way [*that*] sustainability or structure, or all of these things, get assimilated.’ (Participant 2:1)

### Teaching strategies

The different ways that lecturers choose to teach issues of disability to their students, and how disability is presented, emerged as the fourth theme. Out of all the various strategies employed to include disability in the curricula in all six faculties, discussion or workshop was the most utilised strategy, followed by disability as part of a classroom lecture – people with disabilities were also invited to lecture (see Table 4 for other strategies). A participant from the Faculty of Commerce shared that he asks students to look around the classroom and identify possible barriers to participation for students with disabilities in the class, to generate discussion on disability.

**TABLE 4:** Teaching strategies.

Variable	Health Sciences	EBE	Humanities	Law	Commerce	Science
Classroom lecture, seminars and workshops, Course/module	19	6	8	3	3	1
Collaborations with disabled speakers	1	0	1	0	1	1
Reflexive journaling	3	0	0	0	0	0
Policy discussions	2	1	0	1	0	0
Simulations	0	1	1	0	0	0
Peer learning	0	0	1	0	0	0
Disability related articles	0	0	1	0	0	0
Awareness creation and advocacy	1	0	1	0	0	0
Use of film/ tutorials	0	0	1	0	0	0
Group work/ projects / experiential or practice learning	19	4	3	0	0	1
Ad hoc manner	6	1	0	1	2	0

In the Department of Dance (Faculty of Humanities), various journal articles and readings on disability are provided to showcase current disability debates at the postgraduate level. A participant from the Department of Education simulates an inclusive classroom with students; and Diversity Studies^1^ invite people with disabilities to present seminars and workshops to their students. The African Gender Institute (AGI) introduces disability in the classroom through a movie, a lecture, and tutorial discussions.

‘What I must point out is this: that in our tutorial sessions which we have apart from the lectures, students get an opportunity to actually flesh these things out. Because obviously, in a class of – what, 250? 260? – not everybody‘s going to feel brave enough to say how they feel. So what we tend to do is, for our tutorials, there’re probably about fifteen to eighteen in a group.’ (Participant 3:4)

However, the most employed strategies are practice or experiential learning, and classroom discussions. The Transport Division of the Faculty of Engineering and the Built Environment has recorded very good student outcomes from using experiential learning:

‘Because they all go out with instruments to measure grades, they use the wheelchairs to look at ramp gradients, turning circles, the height of buttons to push on lifts, and all of that kind of thing. So it's an enormously powerful exercise. And the type of thing that they write in the course of evaluations is: ‘This was a mind - blowing experience, this has changed my perception of the world.’ (Participant 2:4)

In the Faculty of Health Sciences, the Division of Nursing and Clinical Skills Unit employs practice learning and community engagement as approaches for including disability in their curriculum; similarly to the Department of Health and Rehabilitation Sciences and the Department of Psychiatry, where the case study approach is used. Although the major focus is on impairment and the burden of disease, students begin to interrogate their own reactions to disability and grapple with environmental, socioeconomic and personal factors that create a disabling context for an individual with an impairment:

‘We look at the home, social and occupational environment. We take the students out to practise good psychiatric examination. We use the biopsychosocial model. We teach them how to adjust the environment to help the patient to be functional.’ (Participant 1:18)

The notion of building a more inclusive society is also promoted. The responses from the Division of Information Systems (Faculty of Commerce) suggest that often inventions made for people with disabilities benefit society as a whole:

‘The [*computer*] mouse, which we all use today, was initially designed for people who couldn’t use a keyboard – in other words, who were disabled – and now everybody uses it.’ (Participant 5:1)

Emphasising the need to interrogate our understanding of disability, the following section discusses the study findings related to factors that influence disability inclusion in HEI curricula.

## Discussion

The understanding of disability and the focus on and strategy of inclusion have mostly been influenced by personal interest and disciplinary requirements. These two factors are the main motivations for disability inclusion at UCT among academics who participated in the study. In other words, the extent and manner of disability inclusion is determined by the level of interest the lecturers have in disability. Ramsden (2003) notes that teaching is often influenced by the lecturer's belief system and the values they bring to the teaching and learning experience. Many factors go into being a lecturer, including the ability to critically examine and deconstruct the different ‘selves’ that lecturers are in the teaching space (Ruth 2014). Toohey (1999) refers to these as ‘curriculum ideologies’ – the ideologies that influence the curriculum come out of our personal beliefs and experiences, as well as our understanding of the discipline we are in. So there needs to be critical reflection on the disciplinary frameworks used to understand disability, as this has implications for disability inclusion.

The large amount of content to be covered in the curriculum (and the need to fulfil disciplinary obligations, in the Faculties of Health Sciences, Engineering and the Built Environment, Law, and Commerce) means that often, the focus is on the impairment. The societal and attitudinal causes of disability receive less attention, and are often included only if there is a link to the ongoing classroom discussion. We found that disability is more readily located in the enacted and life curricula, where lecturers with a specific personal interest in the topic include disability in their day-to-day teaching and classroom engagement. Disability should be included in the intended curricula of the various departments as a transdisciplinary issue for effective integration, like other issues of diversity such as race, gender, age and socioeconomic status.

During the interviews, participants were asked to indicate whether they teach on other issues of diversity. Many of them responded that besides disability, they include at least one issue of diversity as an aspect of their intended curriculum and teaching – though Smith *et al.* (2011) found that disability is often not included in the same structured manner as other diversity issues.

One reason for this minimal or inadequate inclusion could be that disability is often seen as a medical or impairment issue (Lellis 2011), as was the case, in this study, in the Faculties of Health Sciences and Engineering and the Built Environment. Disability is not always perceived to be an important part of knowledge acquisition or knowledge construction for students in these disciplines, or in other disciplines. This understanding of disability can result in the ‘invisibility’ of disability (Erevelles 2011).

The findings indicate that there is a need for a framework within the institutional culture and overarching curriculum structure that makes obvious the relevance of disability to shaping our thinking as a society. Inclusion is a complicated process at best, and requires institutions and curricula to create a structure and a system that allow for a culture and practice in which all barriers and opposition to participation may be identified and removed (Tressou, Mitakidou & Karagianni 2007). Giving academic staff sufficient support to include disability is critically important. Where disability is devalued and kept out of the intended curriculum, such support is less likely to occur.

The relative absence of disability inclusion in the curriculum reflects that its relevance is not valued. Toohey (1999) identifies certain factors that influence curriculum content and the value placed on that content. Some of the factors are: our view of the knowledge, the learning process, learning goals, choosing and organising content, and the resources available, including time allocation. The time allocated in the curriculum and the time spent in preparation and availability to students are identified as the major determinants of learning. Not enough time is given to preparation and teaching on disability in the curriculum, as evidenced by the ad hoc inclusions mentioned by many respondents. This practice indirectly reflects the value placed on disability inclusion.

Where disability inclusion does happen, staff often give first priority to their disciplinary requirements; which might lean more towards impairment than to the social discourses of disability. There is a need to create a space within the intended curriculum that explores the socio-cultural aspect of disability in teaching and learning. This learning would support a variety of issues, debates and voices that reveal to students the knowledge community of disability and its discourses, participants and values (Northedge 2003). When no time is planned for structured and detailed interaction, and disability is included in an ad hoc manner, this learning and influence may be undermined.

Including disability in the curriculum discourse would help lecturers, students and researchers to rethink what they consider to be standards of normalcy in society, and to challenge and critique curriculum development and theory. Making disability visible would contribute to creating role models that could show a more positive aspect of disability.

Showcasing the positive aspects of disability was a relevant part of inclusion identified by respondents. Life curricula occur when the lecturer or facilitator is in class with students, engaging with and discussing the subject matter or topic of the course or module. So this ongoing engagement that occurs during life curricula is a very good space for ‘opening students up’ to the ‘humanness’ of disability. In fulfilling the life curriculum, Diversity Studies, Dance, Information Systems and Transport invite people with disabilities to their classes, not only to create awareness and showcase role models, but to enrich the curriculum with critical discussions and debates regarding disability.

As the curriculum is the centre of teaching and learning in HEIs, departments and academics need time to deliver well-planned courses for effective teaching that encourages student learning (Ramsden 2003) and may influence societal change. The inclusion of disability beyond the enacted and life curriculum into the intended curriculum is necessary to create an enabling and supportive environment in HEIs where diversity in the curriculum is encouraged.

### Implications

There is a need to create learning spaces where diversity is valued, which can also contribute to the building of an inclusive space for all learners. However, factors such as the physical learning environment, the core requirements of the curriculum or discipline, the teacher's knowledge and personal experiences, and prevailing cultural and systemic support are some of the factors that influence the teaching and learning space (Toohey 1999). When disability is included in an ad hoc manner, students may be left with the impression that disability is not an important or relevant issue for inclusion in their future professional practice and careers. For excellence to prevail, this shortcoming should be addressed as a matter of urgency.

Time allocation in teaching and learning is important, and disability should be allocated a time and space within the curriculum, across disciplines, along with other issues of diversity. HEIs continue to be the main source of knowledge production and distribution (Atuahene 2011), and with the interdisciplinary nature of disability studies, great strides could be taken towards creating a more inclusive society, as all would be made aware, at an early stage, of the need to do so.

## Conclusion

This study has revealed that there is growing interest from academic staff in including disability; but an overcrowded curriculum has presented challenges to such inclusion in teaching and research. The sense of commitment from the staff who strive to include disability in their own way is commendable. This commitment is an indicator of the necessity to further explore ways and means of providing institutional support for disability inclusion. If students do not encounter disability debates and interrogate notions of difference, normalcy and disability inclusion while in undergraduate studies, they may never have another opportunity to do so.

It is probable that many students will encounter disability; but it is how the students work with the theory and practice that will change the framework of thinking to impact society positively. The Disability Studies Division is a resource that is well positioned to foster the drive for interdisciplinary, innovative, disability-related teaching and research in Africa. This drive contributes to curriculum transformation and supports lecturers with current debates, voices and participatory means of influencing pedagogy regarding disability inclusion across disciplines.

## Footnotes

1 At the time of writing, the Diversity Studies programme (Faculty of Humanities) is no longer offered at the University of Cape Town.
